# Rehabilitation care planning on a digital communication platform for patients with a work disability: protocol for the RehaPro-SERVE feasibility study

**DOI:** 10.1186/s40814-021-00957-2

**Published:** 2021-12-21

**Authors:** Veronika van der Wardt, Hannah Seipp, Annette Becker, Catharina Maulbecker-Armstrong, Rebecca Kraicker, Annika Schneider, Andreas Heitz, Ulf Seifart

**Affiliations:** 1 Department of General Practice/Family Medicine, Philipps-University of Marburg, Karl-von-Frisch-Straße 4, 35032 Marburg, Germany; 2 Faculty of Health Sciences, University of Applied Sciences Central Hesse (Technische Hochschule Mittelhessen), Wiesenstraße 14, 35390 Giessen, Germany; 3 Hospital Sonnenblick, German Pension Insurance, Amöneburger Straße 1-6, 35043 Marburg, Germany

**Keywords:** Rehabilitation, Primary healthcare, Randomised controlled trial, Patient care planning, Case management, Online systems, Feasibility study, Return to work

## Abstract

**Background:**

Long-term disability to work is a risk factor for a permanent reduction in income. Rehabilitation care can support people to return to work. In Germany, rehabilitation care to return to work is mostly provided in specialised clinics. The aim of the Rehapro-SERVE study is to reduce work disability days by facilitating rehabilitation care planning using a digital communication platform. To investigate the feasibility, we will test the implementation of the digital platform and evaluate the study procedures. The Rehapro-SERVE study is funded by the German Federal Ministry of Labour and Social Affairs (BMAS) (grant number: 661R0053K1).

**Method:**

The feasibility study includes a two-armed unblinded block randomised controlled study (RCT) without follow-up assessments as well as an interview study. Participants for the RCT (*n* = 16) are primary care patients with a minimum of 4 weeks of absence from work due to musculoskeletal, oncological or psychological conditions and at high risk of early retirement. Eligibility criteria are age 40 to 60 years; minimum of 4 weeks continuous sick leave before recruitment due to musculoskeletal, mental health or oncological conditions; and being at high risk of early retirement. Patients will be recruited from 8 primary care practices in urban and rural areas in Hesse, Germany. Following baseline assessments, patients will be randomised to either digitalised care planning (treatment) or a control group. The digitalised care planning platform will include the patients’ primary care physicians, jobcentres and public health physicians to decide on a tailored return-to-work programme. The collaboration will be supported by a case administrator and, if considered beneficial, a social worker for the patient. An interview study will evaluate the acceptability of the study procedures and the intervention.

**Discussion:**

The use of a digital communication platform enables stakeholders to exchange information and discuss rehabilitation care planning in a timely fashion. The results of the feasibility study will lead to the adaptation of study procedures for the main study. The results will support the design and conduct of similar studies including digital applications in primary care or across different healthcare settings.

**Trial registration:**

DRKS- German Clinical Trials Register, DRKS00024207. Registered on 22 March 2021

**Supplementary Information:**

The online version contains supplementary material available at 10.1186/s40814-021-00957-2.

## Background

Multi-disciplinary care planning has been shown to improve functional outcomes for different clinical groups [1, 2]. When there is no regular and frequent opportunity for clinicians to discuss cases in person (e.g. in team meetings), digital communication tools can enable efficient communication to initiate and organise care in a timely fashion. Potential benefits of digital platforms for planning and monitoring care have been described in the context of COVID-19 [3] and other health conditions [4, 5], but there is very limited evidence with regard to their effectiveness, in particular, in rehabilitation care.

In Germany, working-age patients can apply to their pension insurance for return-to-work rehabilitation therapy in order to prevent early retirement. In addition to cerebro- and cardiovascular events, the main reasons to qualify for rehabilitation therapy besides diseases of the circulatory system are musculoskeletal issues, cancer-related rehabilitation needs and mental health problems [6]. For the application, patients need to be at “considerable risk” of being unable to continue working, for which they need a medical indication from their primary care or specialist care physician. In-patient rehabilitation care can support the return to work, for example, in cancer survivors and patients with musculoskeletal disorders [7, 8]. If the application for rehabilitation care is approved, therapy will start when rehabilitation places are available at the patient’s chosen rehabilitation centre. In Germany, 84% of adult patients whose rehabilitation application is approved will be admitted to in-patient rehabilitation centres [9]. Therapy programmes will then be developed by physicians at the rehabilitation centre and usually last about 3 to 4 weeks [10]. The therapy treatments can include medical rehabilitation or participation in working life (e.g. vocational training) [11]. In addition, patients can qualify for return-to-work support from their local jobcentres, e.g. additional job skills training, environmental support for their workplace and mobility support.

Currently, the application and decision procedure for rehabilitation care are completed on paper, and the physicians (public health physicians at pension insurances, primary or specialised care physicians) involved in the process do not discuss the treatment. Procedural problems and lack of communication are considered the reasons for delays in starting the rehabilitation or return-to-work programme [12, 13]. In addition, current insurance guidelines do not allow for a flexible approach to implement individual rehabilitation treatments. Therefore, a complex intervention using a digital communication platform combined with a flexible clinical decision-based approach for rehabilitation treatments will be used to facilitate rehabilitation care planning. As previous studies showed, recruiting primary care physicians for the use of digital applications and integrating online-based tools into primary care practices is complex and time-consuming [14]. For a larger randomised controlled trial, study integration into existing processes should be optimised. Consequently, the aim of this feasibility study is to examine the feasibility, implementation and acceptability of study procedures.

## Method

### Design

The study will include a parallel two-arm unblinded block randomised controlled trial (RCT) to explore the feasibility of study procedures in a primary care setting. The study will not include follow-up assessments as it is assumed that these can be completed. In addition, an interview study with primary care physicians and patient participants will be completed to explore the acceptability of study procedures. The feasibility study is expected to start in July 2021 and should be completed within 4–6 months. This article follows the SPIRIT Statement (Supplemental Material A) and CONSORT extension to pilot and feasibility trials (Supplemental Material B) [15, 16]. Ethics approval was granted from the Faculty of Medicine Ethics Committee at the Philipps-University Marburg (study reference 164/20).

### Eligibility criteria for participants

The feasibility study will include 8 primary care physicians and 16 patient participants.

#### Primary care physician participants

Inclusion criteria: primary care physicians will be recruited from practices around the city of Frankfurt (urban area) and the region of middle and northern Hesse (rural areas), Germany.

Exclusion criteria: unwillingness to recruit 2 patients for the feasibility RCT; unwillingness to participate in an interview.

#### Patient participants

Inclusion criteria: age 40 to 60 years; minimum of 4 weeks continuous sick leave before recruitment due to musculoskeletal, mental health or oncological conditions; high risk of early retirement (scores ≤ 36 on the Work Ability Index [17, 18]).

Exclusion criteria: application for rehabilitation treatment in progress; being retired, receiving a retirement pension or disability benefits; being covered by private health insurance; working as a civil servant; permanently living abroad; unable to speak and read sufficient German to read the study information and participate in the interview; and health issues that prevent participation in rehabilitation therapy.

### Recruitment

#### Primary care physicians

In each area (Frankfurt and middle/northern Hesse, Germany), four physicians will be recruited (1) to identify potential patient participants, (2) to support patient participants’ rehabilitation treatment using the digital communication platform and (3) to participate in interviews to provide feedback on the study processes and their experience using the digital communication platform Cankado [19]. Primary care physicians will be selected to include a range of practice characteristics (urban/rural, individual/group practices, size of patient list). The study team will contact primary care practices aligned to a primary care research network. If interested, the physicians will receive written study information, and an appointment will be arranged to discuss the study, sign informed consent and complete a short questionnaire about practice and physicians’ characteristics. Primary care physicians will then receive training for the use of the digital communication platform.

#### Patient participants

Using the eligibility criteria above, each primary care physician will identify potential patient participants from their practice database, as well as during patient visits until two patients have been recruited. Interested patients will receive written study information including contact details for the study team. Patients can either contact the study team themselves or provide written permission to be contacted by the study team. Eligibility criteria will be checked by telephone. If eligible, an appointment with a research assistant will be made.

The patients can decide whether the appointment takes place in person or via videoconference. During this appointment, the study will be discussed, and patients have the opportunity to ask questions. If they decide to participate, they sign an informed consent, complete the baseline assessment and will be entered on the digital platform as data collection will partly be digitalised and partly be completed on paper.

### Randomisation

Following a baseline assessment, patient participants will be randomised individually per computer-generated random sequence into the intervention or control group using a secure web-based application. Programming of the randomisation application allows participant allocation only once, will be logged in a database and cannot be altered. A block randomisation per primary care practice ensures that from each practice, one patient is included in the intervention group and one person in the control group. This allows testing of the randomisation procedures. A member of the research team will inform the patient, the primary care physician and the case administrator about the group allocation.

### Intervention

The complex intervention [20] consists of several components:

1. Support by a case administrator, primary care physicians, public health physicians and if relevant an employee of the jobcentre will discuss and arrange the therapy programme in a case conference on the digital communication platform CANKADO [14]. This will allow them to respond quickly but at a time that is convenient for them without organising a meeting for those involved in the case.
2. An individually tailored and flexibly arranged in- and/or out-patient treatment programme. Depending on the patient’s needs and preferences, existing therapies will be included. The treatment programme can consist of the following components: occupational therapy, physiotherapy, return-to-work support, psychotherapy and work-related educational courses.
3. Patients may receive a therapy programme for which they would not be eligible by pension insurance requirements (e.g. insurance participation period was insufficient).
4. If needed, patients will be supported by a social worker.
5. The therapy programme can include regular treatments as well as a more flexible therapy sequence based on clinical decisions (e.g. physiotherapy before surgery), which is currently not funded by the insurance.

The public health physicians, the case administrators and social workers are employed by the German pension insurance and therefore experienced in return-to-work care planning. The case administrator will facilitate the close cooperation between the members of the digital communication platform by managing information and facilitating the process. If a patient is registered in a jobcentre as unemployed, a jobcentre employee will join the digital communication platform, to offer jobcentre services. Patients are not involved in the case conference. The Cankado platform [19] is encrypted and secured; only the persons involved in the respective case management have access to the case conference.

Primary care physicians, public health physicians and, if relevant, jobcentre employees will be notified when participants have been entered into the digital platform for the intervention group in order to alert the physicians that action is needed. The primary care physician will then add relevant information from the participant’s medical history on the platform. The members of the digital communication platform will review the information to discuss treatment options. The primary care physician will discuss the recommended treatment with the patient, and if the patient agrees, the case manager will organise the programme.

If the physicians decide that a participant would need additional support to complete the rehabilitation programme, a social worker will support the implementation of the programme by assisting the patient participant. This can include reminding them of appointments, arranging transport to therapies or help them to plan absences from the family.

The discussions and treatment decisions on the communication platform will continue until the physicians consider the treatment completed. The frequency of communications will depend on the patient participant’s needs and therapy response. Therapy engagement will be monitored by the case administrator; treatment success will not be monitored as part of the intervention but evaluated in the follow-up assessments of the main RCT. The intervention description based on the TIDieR checklist [21] can be found in supplement C.

### Control

Participants in the control group will receive treatment as usual (i.e. their primary care physician stays their contact for medical purposes, and they can apply for return-to-work rehabilitation in the usual way). Their treatment will not be organised using the digital communication platform or supported by a case administrator.

### Outcomes

#### Baseline assessment

The baseline assessment will include the following:

Primary care physicians:

- Demographics (gender, age, work experience, hours of work per week)
- Practice characteristics (practice size, location (urban/semi-urban/rural) and type (individual/group practice))

Patient participants:

- Demographics (gender, age, household size, years of education)
- Employment characteristics (employment status, hours of employment per week)
- Job satisfaction (based on a 0–10 Likert scale)
- Number of days of sick leave in the last 6 months before baseline assessment
- Reason for sick leave (musculoskeletal, mental health or oncological)
- Depressive symptoms (Hospital Anxiety and Depression Scale (HADS-D), German version [22], scores from 0 to 21 each on the anxiety scale and depression scale)
- Work ability (Work Ability Index (WAI), German version [18, 23, 24], scores from 7 to 49)
- Health-related quality of life (Short Form 36 Health Survey (SF-36), German version [25, 26], scores from 0 to 100)

#### Process evaluation

In addition, the following process evaluation data will be recorded:

- Duration of recruitment (from the opening of the study to consenting second participant in each practice; in days)
- Withdrawal rates
- Duration baseline assessments (for each participant; in minutes)
- Time between baseline assessment and start of the first therapy (for each participant; in days)
- Time between baseline assessment and completion of all therapies (for each participant; in days)
- Treatment recommended on the digital communication platform (only intervention group participants)
- Therapies initiated due to study participation but not funded by the pension insurance (only intervention group participants)

#### Follow-up

The study will not include further data collection points. Participants are of working age and will have completed the baseline assessment. Cognitive deterioration is not expected in this group. Therefore, the ability to complete follow-up assessments is assumed.

**Fig. 1:**
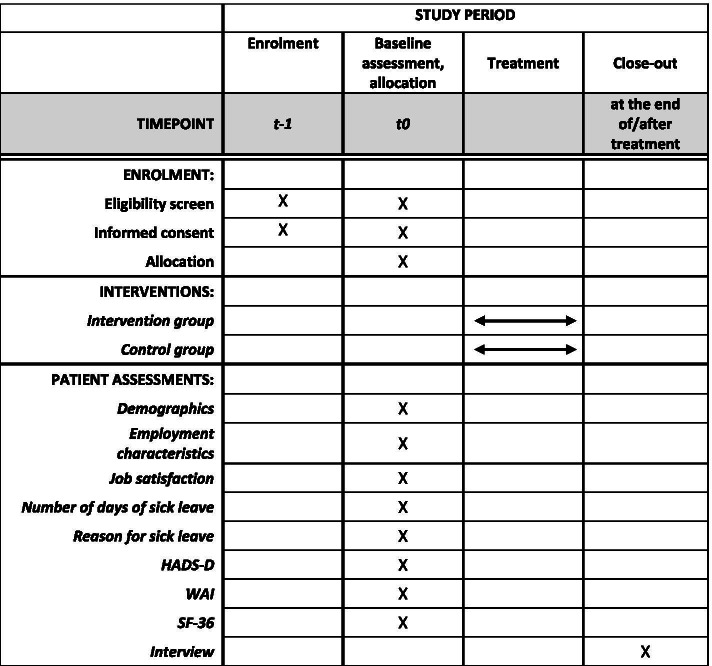
Time schedule of patients’ enrolment, interventions and assessments (SPIRIT flow diagram [14])

The time schedule for patient participants is presented in Fig. 1.

#### Interview study

Participants for the interview study include all primary care physicians (*n* = 8) and patient participants (*n* = 16). Information and consent on the interviews are included in the consent on study participation.

Primary care physicians will be interviewed once they recruited two patients, and the patient randomised to the intervention group has started their therapy programme. The semi-structured interviews will explore the physician’s experience of study procedures (intervention training, recruitment of patient participants, randomisation and use of digital communication platform). Interviews will take place in the primary care practice, by telephone or videoconference.

Patient participants will be interviewed in a place of their choice or by telephone/videoconference at the end of, or after, the rehabilitation therapy. The semi-structured interviews will explore the study procedures (information, recruitment, assessments), the communication with primary care physicians and the patient’s experience with rehabilitation therapies.

The interviewers are experienced in conducting interviews. The interviews are expected to take between 45 and 90 min. They will be recorded and transcribed verbatim.

### Data collection and management

The collection of all primary care physician data will be completed on paper. The collection of patient participant data will be completed digitally in Cankado; only SF-36 and HADS-D will be collected on paper. Data entry will be double-checked. All the other baseline assessments will be completed digitally using the Cankado platform [19] and will be exported and merged with data from the SF-36 and HADS-D questionnaires. Data collected in Cankado will be subject to the European General Data Protection Regulation (GDPR) 2018 and stored on a server in Germany [27]. All research data will be exported from Cankado, password protected and stored on the University of Marburg server with personal data stored separately from research data. Personal data will be deleted at the end of the study, and research data will be deleted 10 years after the study has been completed. A data management plan has been developed based on the European GDPR. Access to the dataset will be limited to the research team of the University of Marburg. Research data will not be accessible for the members of the digital communication platform, except the WAI scores, which physicians will be able to view to include in their treatment decisions.

### Analysis

A descriptive analysis of the quantitative data will be completed. For categorical variables, proportions will be reported; for continuous variables, means, standard deviations and ranges will be presented. Missing data will be reported as part of the completion rates. Data for primary care physicians and patient participants will be reported separately. All quantitative analysis will be completed in the statistical software R [28].

Qualitative interview data will be analysed using the thematic analysis approach outlined by Braun and Clarke [29]. After familiarisation with the data, a researcher will develop preliminary codes, which will be checked and discussed with the research group. Initially, a theory-driven approach will be used based on the questions regarding the study procedures, communication with the primary care physician and the patient’s experience with the rehabilitation therapies. An inductive analysis approach will be used to develop the themes within the framework of these questions. The initial coding will be reviewed within the research team. Themes, sub-themes and relationships between those will be developed and refined until clear definitions of the themes have been achieved. The analysis will be managed using MAXQDA [30].

## Discussion

Decisions regarding return-to-work therapy require the input of different clinicians who currently either correspond by fax and post or not at all. In addition, valuable information about the patient’s medical history and circumstances is often not being considered given the limited communication between the stakeholders. The digital communication platform will enable stakeholders to exchange information, discuss treatment options and develop a treatment plan in a timely and coordinated fashion. Furthermore, the intervention will allow clinicians to prescribe therapies more flexibly based on their clinical decisions but outside the insurance-funded programme. The combination of using digital communication to come to better informed, coordinated and timely therapy decisions, the flexible timing of therapy components and the option to support difficult to engage patients with a personal care manager are the key components of this complex intervention.

This feasibility study is intended to provide information on how the study procedures can be integrated well into clinical processes, as previous studies emphasised the need for adaptation especially in primary care [31]. Primary care physicians welcome communication applications for patient care [32], but a survey indicated concerns regarding data security, time expenditure and implementation difficulties into the daily practice research [33]. The results of this feasibility study will be published in scientific journals and study reports for the funder. They can support similar studies including the use of digital applications in primary care as well as digital communication across different healthcare settings.

### Limitations

The feasibility study will not be blinded nor include follow-up assessments. Blinding to the outcome assessments and data analysis by an independent statistician blinded to group allocation are planned for the main randomised controlled trial. Follow-up assessments were not considered a feasibility issue in this population and therefore are omitted. This, however, will limit the accuracy of the withdrawal rates estimation for the follow-up assessments of the main RCT, which will be obtained from other in-patient rehabilitation trials. A case administrator for the digital platform will be included, e.g. to notify clinicians that action is required, or to check if therapy components are regular prescriptions (i.e. funded by the insurance) or flexible (and therefore funded by the research project). If the intervention is effective and the digital platform implemented in clinical practice, a further digitalisation of the processes is expected. Another limitation is the block randomisation, for the feasibility into blocks of 2 patient participants. After randomisation of the first patient, the allocation of the second participant will be obvious to the primary care physicians who are also responsible for the recruitment in their practice. However, from a feasibility perspective, it was considered important to recruit a number of primary care practices from both regions (urban Frankfurt and rural middle/northern Hesse) and to test the randomisation procedures as they are planned for the main study albeit then with larger blocks. For the decision to use block randomisation, the advantages (potential bias due to treatment allocation) and equally distributed workload for participating primary care practices, outweighed the potential selection bias due to the allocation of the last participant being known [34]. In addition, reasons for sick leave (musculoskeletal, mental health or oncological) will be recorded, but there is no recruitment requirement to include a specific proportion of each.

## Conclusion

The study will test the feasibility and acceptability of recruitment, baseline assessment and implementation of the digital communication platform to improve the application and decision procedure for in-patient return-to-work rehabilitation. Based on the results, the design of the subsequent RCT will be adapted.

## Supplementary Information

**Additional file 1.** SPIRIT 2013 Checklist: Recommended items to address in a clinical trial protocol and related documents.

**Additional file 2.** CONSORT 2010 checklist of information to include when reporting a pilot or feasibility trial.

**Additional file 3.** Intervention description based on TIDieR checklist.

## Data Availability

Not applicable as the article describes no results.

## Abbreviations

BMAS: “Bundesministerium für Arbeit und Soziales” German Federal Ministry of Labour and Social Affairs
DRKS: “Deutsches Register Klinischer Studien” German Clinical Trials Register
HADS-D: Hospital Anxiety and Depression Scale, German version
WAI: Work Ability Index
SF-36: Short Form 36 Health Survey
SPIRIT: Standard Protocol Items: Recommendations for Interventional Trials
