# Supplementary material for: Rehabilitation care planning on a digital communication platform for patients with a work disability: protocol for the RehaPro-SERVE feasibility study
Source: Pilot Feasibility Stud. 2021 Dec 21;7:221. doi: 10.1186/s40814-021-00957-2 (PMC8688904; doi:10.1186/s40814-021-00957-2)
Supplement: Supplementary file 3 — Additional file 3. Intervention description based on TIDieR checklist. [file 40814_2021_957_MOESM3_ESM.docx]

**Supplemental Material C:**

**Intervention description based on TIDieR checklist^^[[1]](#footnote-1)^^**

**Article**: Rehabilitation care-planning on a digital communication platform for patients with a work disability: protocol for the RehaPro-SERVE feasibility study.

**Authors**: Veronika van der Wardt, Hannah Seipp, Annette Becker, Catharina Maulbecker-Armstrong, Rebecca Kraicker, Annika Schneider, Andreas Heitz, Ulf Seifart

| Name | RehaPro-SERVE – digital communication platform |
| --- | --- |
| Why? (rationale) | The aim is to improve rehabilitation care planning to reduce sick leave due to musculoskeletal, psychological or oncological health conditions though:  1) Support by a case administrator, primary care physicians, public health physicians and if relevant an employee of the jobcentre will discuss and arrange the therapy programme in a case conference on the digital communication platform. This will allow them to respond quickly but at a time that is convenient for them without organising a meeting for those involved in the case.  2) an individually tailored and flexibly arranged therapy programme. This can include the following components: occupational therapy, physiotherapy, return-to-work support, psychotherapy and work-related educational courses;  3) patients may receive a therapy programme for which they would not be eligible by pension insurance requirements (e.g., insurance participation period was insufficient);  4) if needed, patients will be supported by a social worker;  5) the therapy programme can include regular treatments as well as a more flexible therapy sequence based on clinical decisions (e.g. physiotherapy before surgery), which is currently not funded by the insurance. |
| What? (procedure) | Primary care physicians, public health physicians and, if relevant, jobcentre employees will be notified when participants have been entered in the digital platform for the intervention group in order to alert the physicians that action is needed. The primary care physician will then add relevant information from the participant’s medical history on the platform. The members of the digital communication platform will review the information to discuss treatment options. The primary care physician will offer the recommended treatment to the patient.  If the physicians decide that a participant would need additional support to complete the rehabilitation program, a social worker will support the implementation of the programme by assisting the patient participant. This can include reminding them of appointments, arranging transport to therapies or help them to plan absences from the family. |
| Who provided? | The digital communication platform will facilitate rehabilitation care planning between the public health and the primary care physician. In addition, the case administrator and, if needed, a jobcentre employee will be included. |
| How? | The discussions will take place online within the communication platform. Required documents (e.g., patient records) can be added shared electronically. |
| Where? | The intervention will take place online on the digital communication platform Cankado. |
| When and how much? | The communication will commence, and initial decisions will be made once the patient has been entered onto the platform. Rehabilitation care planning will continue until the physicians consider the treatment completed. |
| Tailoring | Rehabilitation care therapy will be tailored to the patients’ needs depending on health condition and patient preferences. |
| How well? (adherence/fidelity) | The feasibility study will record adherence and fidelity data (timelines for treatment commencement and treatment completion, treatment recommended, therapies initiated due to study participation but not funded by the pension insurance). |

1. Hoffmann TC, Glasziou PP, Boutron I, et al. Better reporting of interventions: template for intervention description and replication (TIDieR) checklist and guide. BMJ 2014; 348: g1687. [↑](#footnote-ref-1)
